# Lateral Squats Significantly Decrease Sprint Time in Collegiate Baseball Athletes

**DOI:** 10.3390/sports4010019

**Published:** 2016-03-07

**Authors:** Jason B. White, Trevor P. Dorian, Margaret T. Jones

**Affiliations:** 1 Health and Human Performance, George Mason University, Manassas, VA 20110, USA; jwhite35@gmu.edu; 2 Exercise Science and Sport Studies, Springfield College, Springfield, MA 01109, USA

**Keywords:** agility, complex training, lower body strength, postactivation potentiation, speed

## Abstract

The purpose was to examine the effect of prior performance of dumbbell lateral squats (DBLS) on an agility movement-into-a-sprint (AMS) test. Twelve collegiate, resistance-trained, baseball athletes participated in three sessions separated by three days. Session One consisted of AMS baseline test, DBLS 5-RM test, and experimental protocol familiarization. Subjects were randomly assigned the protocol order for Sessions Two and Three, which consisted of warm up followed by 1-min sitting (no-DBLS) or performing the DBLS for 1 × 5 repetitions @ 5RM for each leg. Four minutes of slow recovery walking preceded the AMS test, which consisted of leading off a base and waiting for a visual stimulus. In reaction to stimulus, subjects exerted maximal effort while moving to the right by either pivoting or drop stepping and sprinting for 10 yards (yd). In Session Three, subjects switched protocols (DBLS, no-DBLS). Foot contact time (FCT), stride frequency (SF), stride length (SL), and 10-yd sprint time were measured. There were no differences between conditions for FCT, SF, or SL. Differences existed between DBLS (1.85 ± 0.09 s) and no-DBLS (1.89 ± 0.10 s) for AMS (*p* = 0.03). Results from the current study support the use of DBLS for performance enhancement prior to performing the AMS test.

## 1. Introduction

The importance of lower body muscular power to performance in sporting activities is well documented [1,2,3] Therefore, training techniques designed to improve lower body power are of interest, and methods such as plyometrics and complex training are commonly employed [4,5,6]. These training methods utilize exercises similar to the movements of sporting activity in order to provide sport specific enhancements and improve power development.

The response of skeletal muscle to specific stimuli is a function of the prior contraction history [7]. Complex training, a method that involves performing a moderate to heavy resistance exercise as a conditioning contraction followed by a lighter-resistance ballistic activity, has been shown to elicit greater lower body power production in subsequent explosive movements [8,9]. An example of complex training is performing a squat as the heavy strength exercise, followed by explosive vertical jumps. Acute performance enhancement found in power movements following a conditioning contraction exercise, such as a heavily loaded resistance exercise or maximal voluntary contractions (MVC), is likely due to post-activation potentiation (PAP) [10,11,12,13,14], which is defined as an increase in muscle function following a preload stimulus [7]. The application of complex training to elicit PAP, both acutely and in training programs, has long been thought to improve lower body power [4,15,16,17]. Both PAP and neuromuscular fatigue are stimulated by the same factors [18] and can occur simultaneously [19], but the greatest motor performance occurs with minimal neuromuscular fatigue [7]. It may be for this reason that the numerous studies that have examined complex training and PAP have pursued acute identification of the optimal load or the timing after loading where peak power production occurs. Previous research with resistance-trained athletes indicates that the optimal load for complex training may be achieved using MVCs [11] or 60%–85% 1-RM [20] and 3 to 8 min rest between the heavy strength exercise and the ensuing explosive movement [20,21]. However, the effect of complex training on PAP has been shown to vary with the individual’s training status, exercise intensity, exercise volume, and length of rest period between the strength exercise and the explosive activity [20,22]. Further, it has been suggested that the interplay between a subject’s individual characteristics and the protocol design (e.g., strength and power exercise selection) may have an important effect on the extent of potentiation elicited [23]. 

Strength is critical to developing force rapidly and is integral to the baseball skills of batting, throwing, and running [24], and agility movements (e.g., drop step, pivot, shuffle) are important when reacting to a stimulus (e.g., fielding, leading off of a base). Previous methodology to induce PAP has utilized dynamic movements, such as squat [13], bench press [25], Olympic lifts [26], loaded drop jumps [27], as well as isometric MVC [11,28]. Little research exists to indicate how PAP induced by lateral movements acutely impacts lower body power development. Further, because most studies have used the squat and vertical jump as the conditioning contraction exercise and ensuing explosive movement, respectively, little is known about the impact of loaded lateral strength exercises on explosive movement and power production. Yet, sports such as baseball, basketball, hockey, and football require as much as 50%–90% agility movements in a lateral direction [29]. Additionally, little research has been completed analyzing methods to improve the first stride in a frontal plane following a stationary athletic position. Therefore, the purpose of the current study was to examine the acute impact of dumbbell lateral squat (DBLS) exercise on an agility movement-into-a-sprint (AMS) test in collegiate baseball players.

## 2. Material and Methods

### 2.1. Subjects

Twelve (*n* = 12) National Collegiate Athletic Association (NCAA) Division-III resistance-trained baseball position players volunteered to participate in the current study. All subjects were proficient weight lifters and had ≥ 1 year of formal strength and conditioning training experience. All subjects were medically cleared for intercollegiate athletic participation, had the risks and benefits explained to them beforehand, signed an institutionally approved consent form to participate, and completed a medical history form. The Institutional Review Board for Human Subjects approved all procedures. Exclusion criteria consisted of severe musculoskeletal injuries of the lower body or spinal injuries within 6 months before the start of the study. The subjects were instructed to refrain from lower body exercise for 72 h before each testing session. Also, they were asked to consume an identical diet for the 24 h before each testing session. Testing sessions were conducted during the off-season training period. All subjects in the current study were members of the same baseball team, and were familiar with the DBLS exercise and the AMS test from their participation in the same collegiate strength and conditioning off-season program. Physical characteristics are included in Table 1.

### 2.2. Experimental Design

The purpose of the current study was to investigate the acute effect of DBLS exercise on an agility movement into a 10 yd sprint. A within-subject randomized crossover design was used as subjects completed the AMS test following DBLS exercise and a control condition (no-DBLS) without DBLS exercise. In both the DBLS and no-DBLS conditions, four minutes of slow recovery walking preceded the AMS test.

### 2.3. Procedures

#### 2.3.1. Session One: 5-RM DBLS Testing and Protocol Familiarization

Session One consisted of completing the informed consent and medical history forms, familiarization with the 10-min supervised standardized warm-up (*i.e.*, five minutes of stationary cycling at 70–80 RPM with a 0.5-kg load and five min of dynamic flexibility exercises), application of reflective markers, baseline AMS test, and 5-RM DBLS test. Prior to testing and familiarization, subjects’ height and body mass were determined to the nearest 0.1 cm and 0.1 kg, respectively, using a stadiometer (Tanita; Arlington Heights, IL, USA) and self-calibrating digital scale (Tanita; Arlington Heights, IL, USA) with subjects in sock feet.

#### 2.3.2. Sessions Two and Three: Experimental Protocol

Seventy-two hours after Session One, subjects returned to the laboratory at the same time of day on two separate occasions, each separated by seventy-two hours, to perform two randomly ordered experimental trials (DBLS, no-DBLS). Athletes were randomly assigned to the DBLS or no-DBLS protocol. In Session Three, which took place 72 h later, the subjects switched protocols. On the day of each experimental trial, subjects arrived having refrained from lower body resistive exercise since the previous experimental trial. After a supervised, standardized, 10-min warm-up identical to that performed prior to testing during session one, subjects performed DBLS exercise of 1 × 5 repetitions @ 5-RM for each leg or sat quietly for 1 min, depending upon their assigned protocol. The DBLS exercise was performed according to the methods described for the 5-RM test. However, both legs were trained. After the left knee flexed to meet the proper squat depth five consecutive times, the right leg followed. Following four minutes of slow recovery walking subjects performed the AMS test.

#### 2.3.3. 5-RM DBLS Test

**Figure 1:**
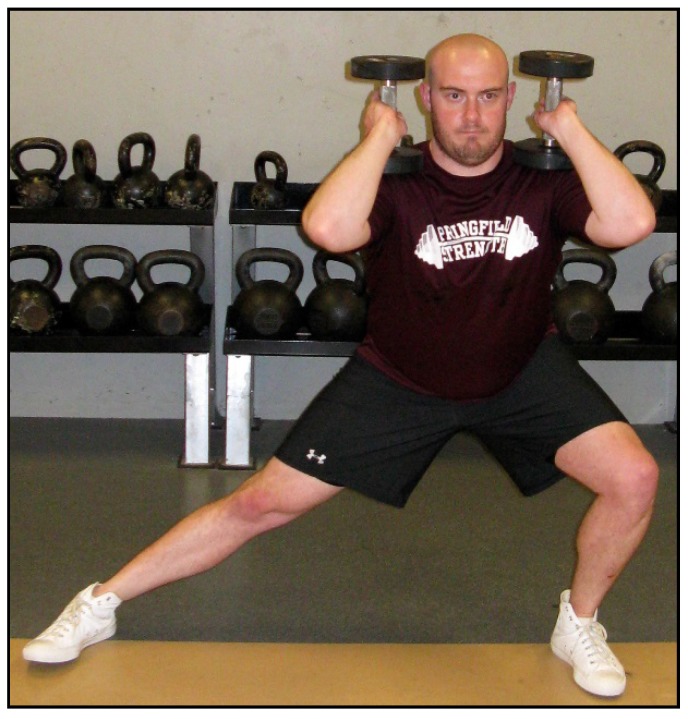
Mid-range of the DBLS exercise.

Lateral squat [30] strength was assessed for the right leg with a 5-RM test using dumbbells. Briefly, subjects completed a 10-min whole body warm up followed by supervised warm up sets for the DBLS test. While performing the DBLS, the athlete assumed a stance approximately twice that of shoulder width (Figure 1). Each subject’s foot placement was measured and recorded. The pelvis was tilted posteriorly and the subject performed a downward squatting motion until the anterior thigh was parallel to the floor. When the subject reached parallel squat depth, a sound was emitted from the safety squat beeper (Bigger, Faster, Stronger, Inc., Salt Lake City, UT, USA), which had been placed anteriorly on the thigh of the right leg [24]. The beeper location was noted for each subject and repeated for subsequent experimental sessions. In the down position of the DBLS, both heels remained in contact with the floor. The down position was held for one second. Upon completion of the down position, the subject rose to the upward position with full extension in the hips and knees, while keeping the feet in contact with ground. Dumbbells were rested upon the anterior deltoids of the subject in a front squat hold position. A timed rest of three minutes was taken before each maximal effort set. Weight was increased based upon the performance of the previous attempt, and the subject continued to perform sets of five repetitions until failure or until it was determined that he could no longer perform the DBLS safely with proper form. After two failures, testing was stopped, and the best lift for 5-RM was recorded.

#### 2.3.4. AMS Test

The AMS test was conducted in the laboratory following the DBLS or no-DBLS exercise. All subjects performed a 4-min slow walking recovery [21] prior to the start of the AMS test. The AMS consisted of performing a 10-yd sprint from a base runner’s stance for the starting position. Ten reflective markers were applied to the body: left and right anterior superior iliac spine, center of the left and right patella, left and right mid-thigh, distal anterior portion of the left and right tibia, medial malleolus on the right leg, and lateral malleolus on the left leg. Individual anatomical locations of marker placements were located and marked on each subject with semi-permanent ink to enable reproducibility for subsequent measures. Athletes were required to wear compression shorts to clarify movement of the lower extremities for the video analysis. Prior to the initiation of the agility movement, the subject was stationary and had a stance slightly wider than shoulder width, with slight flexion in the knees and hips. Body weight was distributed evenly on the balls of the feet, and both feet were parallel with the sagittal plane. Each subject’s foot placement was measured and recorded. The direction of movement was to the right of the subject in the frontal plane, which is identical to stealing a base in baseball; consequently, athletes were well versed in the movement. The initial movement began upon reaction to a visual stimulus, which was a light emitting diode. The subject exerted maximal effort while moving to the right by either pivoting both feet or drop stepping with the right foot in order to place the feet in a position similar to that of a linear sprint during initial acceleration. The subject maximally sprinted for 10 yd. Each athlete’s choice of a pivot or drop step was noted by the researcher and repeated during subsequent testing sessions.

In AMS testing Sessions Two and Three, the foot contact time (FCT) and stride length (SL) for the Stride 1 and Stride 2, and stride frequency (SF) for Segment 1 and Segment 2 were recorded by a video camera (50 fps, JVC GR-DVL9800 Mini, JVC USA, Wayne, NJ), which was placed perpendicular to the sagittal plane of the subject and 10 yd from the running lane. Markers were placed at 0, 5, and 10 yd on the blank wall that was parallel to the running lane. The same trained researcher analyzed all recordings using two-dimensional video analysis software (Dartfish 5.0 Connect, Fribourg, Switzerland). Timing sensors with single beamed infrared photocells (Brower Timing Systems, Salt Lake City, UT, USA) were placed to capture the time at the 5 and 10 yd locations. 

Test-retest reliability for the AMS was determined from data collected during baseline AMS tests in session one and the subsequent no-DBLS condition, which was either Session Two or Three depending upon the random order assignment of each subject’s no-DBLS condition.

Foot contact time (FCT). The FCT time was defined as the time that each foot was in contact with the ground following initial lower body movement. The initial movement pattern varied based upon how the athletes were taught to steal a base (*i.e.*, drop step *vs.* pivot). Approximately half of the athletes drop stepped with the right foot prior to pivoting and striding with the left. Only FCT prior to a stride was recorded in order to maintain consistency among measures. Athletes repeated their chosen initial movement (*i.e.*, drop step, pivot) for both DBLS and no-DBLS conditions.

Stride frequency (SF). For comparison of SF or number of strides per section of distance, the AMS test was divided into two segments: 0–5 (Segment 1) and 6–10 yd (Segment 2). Using video analysis, SF for each segment was determined from the total number of strides taken during the total time taken to cover Segment 1 and to cover Segment 2.

Stride length (SL). Stride length for Stride 1 and Stride 2 were determined from video analysis and recorded in inches for both experimental sessions. Stride 1 was defined as the distance covered between first two successive ground contacts by the left foot. Stride 2 was defined as the distance covered between next two successive ground contacts by the right foot. 

### 2.4. Statistical Analyses

Normality of data was assessed by the Kolmogorov-Smirnov test of normality, which determined all primary outcome measures of interest to be normally distributed. Foot contact (FCT), stride frequency (SF), and stride length (SL) were analyzed with 2 (condition: DBLS, no-DBLS) × 2 [time (FCT), distance (SL), number (SF)] repeated measures analysis of variance (ANOVA). The AMS test was analyzed by a 2 (condition) × 3 (segment 1, segment 2, total time) repeated measures ANOVA. Bonferroni’s pairwise post hoc analyses examined differences across condition and within testing periods. Effect sizes were calculated and a modified classification system (trivial, 0.0–0.2; small, 0.2–0.6; moderate, 0.6–1.2; large, 1.2–2.0; very large, >2.0; extremely large, >4.0) was used [31]. Test-retest reliability was determined through the intra-class correlation coefficient (ICC). Statistical procedures were conducted using the Statistical Package for the Social Sciences (IBM SPSS Statistics 20.0, IBM Corporation, Armonk, NY, USA). The alpha level was set at *p* < 0.05.

## 3. Results

**Figure 2:**
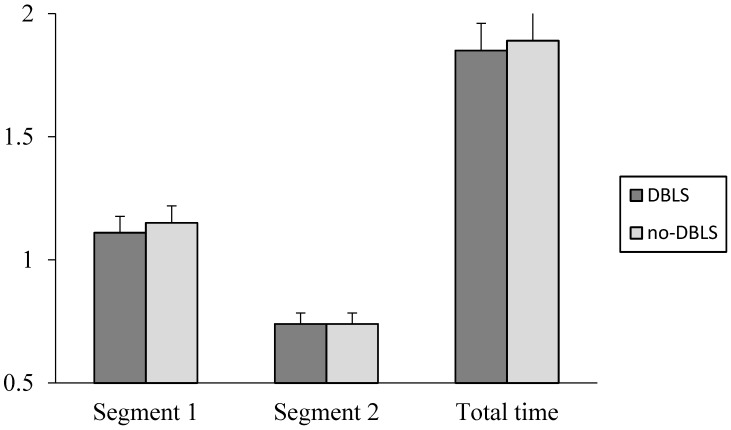
AMS test time (s) for DBLS and no-DBLS conditions. ^#^ significant difference (*p <* 0.05) between total LMS time for DBLS and no-DBLS conditions; * significant difference (*p <* 0.05) between Segment 1 (0–5 yd) and Segment 2 (6–10 yd).

There was no significant interaction between the DBLS and no-DBLS conditions for FCT (*p* = 0.70), SF (*p* = 0.28), or SL (*p* = 0.79) (Table 2). Significant differences were found for FCT (ES = 5.3, *p* = 0.0001) and SL (ES = 1.2, *p* = 0.0004) between Stride 1 and Stride 2. The SF for Segment 2 (6–10 yard) was significantly (ES = 2.2, *p* < 0.0002) less than for Segment 1 (0–5 yard). A significant difference existed between conditions for the AMS test for Segment 1 time (ES = 0.61, *p* = 0.01, DBLS: 1.11 ± 0.06 s; no-DBLS: 1.15 ± 0.07 s), and total time (ES = 0.42, *p* = 0.03, DBLS: 1.85 ± 0.09 s; no-DBLS: 1.89 ± 0.10 s) (Figure 2). There was no significant difference (*p* = 0.414) in overall AMS time between session one (1.90 ± 0.09 s) and the no-DBLS condition (1.89 ± 0.10 s), and the ICC of 0.98 between the two testing sessions was considered strong.

## 4. Discussion

This is the first study to examine whether or not an acute bout of DBLS exercise would enhance agility movement and the subsequent sprint in resistance-trained, collegiate baseball athletes. It was hypothesized that measures of FCT, SF, SL, and AMS time would improve as a result of acute lower body resistive exercise. The overall findings supported a positive effect on the use of resistive exercise on AMS time; however, no difference was found for FCT, SF, or SL across the DBLS and no-DBLS conditions. To our knowledge, no study to date has reported on the effect of complex training on an agility movement-into-a-sprint, FCT, SF, or SL in baseball athletes, yet, baseball is a sport that requires a large percentage of agility movements in a lateral direction [29]. The performance measure (AMS test) in the current study was comparable to the baseball specific movement of stealing a base [32]. It required reaction to a visual stimulus, quick change of direction, and a short burst sprint, all of which are regularly used movement patterns in baseball [33].

The mechanisms responsible for eliciting PAP and the optimal conditions for performance enhancement from complex training have not been clearly defined. The utilization of dynamic strength movements for a conditioning exercise to induce PAP during plyometric exercise has yielded mixed results [10,14,23,34,35,36]. Previous research has demonstrated improved sprint times following single joint and isometric strength exercise [37]. However, the effect of dynamic strength exercise on sprint time is equivocal and varies by individual and rest time [21,22]. Further, the exercise selection and intensity of the conditioning contraction can impact subsequent power production [20]. Complex training has resulted in improved 10-m shuttle run time [38], 10-m [22], 40-m [13], and 100-m [39] sprint times. In contrast, other research has shown no effect on 10-m [13], 30-m [24] and 40-m [26] sprint times.

In the current study, complex training did not elicit a PAP response for the FCT, which was defined as the time that each foot was in contact with the ground following initial lower body movement until the foot completed the stride. The contact time for Stride 1 was significantly lower than Stride 2, irrespective of DBLS or no-DBLS condition. This was likely due to the required movement direction to the right, which was similar to that necessary when stealing a base. As a result, the left foot always completed Stride 1, and the right foot stayed in contact with the ground for a longer period of time. Varying differences in the agility movement patterns (drop step, pivot) were existent, but not the focus of the current study.

Complex training did not elicit a PAP response for the SF. In both conditions the SF for Segment 1 was significantly greater than Segment 2, which is likely a result of the short explosive strides required during the initial acceleration of Segment 1 *versus* the longer strides of Segment 2 as the athlete increased his running speed [40].

The primary purpose of examining SL was to determine change in SL due to PAP; however, the condition (DBLS, no-DBLS) had no effect on SL in the current study. Irrespective of condition, Stride 2 was significantly greater than Stride 1, and this finding was likely due to the specific mechanical tendencies of the movement pattern. The initial agility movement that was performed prior to sprinting was identical to that of a baseball player attempting to steal a base; therefore, the subject was required to move to his right in the frontal plane following a stationary athletic position. After visual examination of the digital recordings of running mechanics it was clear that an initial stride of the subject with the left foot was needed in order to rotate the hips and shoulders to the right and rapidly move the center of gravity so that the momentum of the subject was correctly oriented toward the target. The pivoting limited the initial stride of the left foot. The subject could then make a significantly greater second stride with the left foot because he was facing in the direction of the sprinting destination.

Regardless of condition (DBLS, no-DBLS) the AMS test time for Segment 2 (6–10 yd) was significantly lower than Segment 1 (0–5 yd), which is likely a result of the athlete beginning the AMS test from a stationary athletic stance position. After accelerating through the 5 yd sensor and completing Segment 1, the athlete had created enough momentum to propel himself through Segment 2 and the final 10 yd sensor at a faster speed.

While FCT, SL, and SF, did not differ between DBLS and no-DBLS conditions, AMS Segment 1 and total time were significantly lower (0.04 s) for the DBLS condition than the no-DBLS condition; therefore, the resistive exercise may have induced PAP, which enhanced the sprint time. Segment 1 was 3.6% faster and the total AMS time was 2.2% faster when the AMS was preceded by DBLS. Although the effect size was small, milliseconds can determine whether or not a base runner is safe or out, therefore, such a magnitude of improvement might have noteworthy ramifications during competition. In the current study, the DBLS exercise was followed by a 4-min rest period during which the subjects completed a slow recovery walk prior to performing the AMS test. In previous research reporting a significant PAP effect during sprinting distances of 10-m [22], 30-m [38], 40-m [13], and 100-m [39], the dynamic resistance exercise selection and intensity have varied for the conditioning contraction, but the most effective rest periods have consistently been ≥ 4 min. The design of the current study was unique because it examined the effects of PAP on a ballistic agility movement that led into a sprint. The use of resistance-trained baseball athletes who were familiar with the AMS test and the DBLS in conjunction with the standardization of all warm-up procedures may have also been contributing factors in the positive results of the current study.

We acknowledge some study limitations. First, although all subjects in the current study were resistance-trained collegiate baseball position players, the sample size of twelve was small. A larger sample size may have resulted in other differences between the DBLS and no-DBLS conditions. It has been demonstrated that results from studies examining PAP in relation to performance on field tests are equivocal, in part due to the varied individual response to complex training methods [20,22,26]. Second, a higher grade of video analysis software may have allowed for better discrimination in measures of FCT, SL, and SF.

It is well known that responses to a PAP protocol may vary depending upon the individual’s training background and strength level [14,23,34,36,39]. Future research that examines the effects of dynamic strength movements on varied sprint distances, repeated sprint measures, and the establishment of how long the PAP effect remains are warranted. The investigation of efficiency of various agility movement patterns (e.g., drop step, pivot) for directional change would also be of interest. Further, the effect of implementing such complex training methods between innings during game play would be of interest to the baseball practitioner.

## 5. Conclusions

Results from the current study are applicable to sport and training. Successful skill execution in baseball often requires the development of power over a short period of time. Effective use of PAP (movement specific conditioning exercise, proper rest time) in the form of complex training can enable an individual to train at a greater intensity, therefore, attaining superior gains in power production. Program implementation of complex training, which pairs DB lateral squats with short sprints, may provide an adequate training stimulus for enhancing agility movements and, thereby, improve base running.

## Figures and Tables

**Table 1:** Physical characteristics of baseball athletes.

Physical Characteristic	Mean	*SD*
Age (y)	19.9	1.2
Training experience (y)	3.2	2.1
Height (cm)	175.9	6.6
Body mass (kg)	79.9	10.9
5-RM DBLS (kg)	18.2	4.9

*n* = 12. Year (y); Centimeter (cm); Kilogram (kg); Standard deviation (*SD*).

**Table 2:** Foot contact, stride frequency, and stride length for DBLS and no-DBLS conditions.

Measure	DBLS Condition M ± *SD*	no-DBLS Condition M ± *SD*
Foot Contact Time (FCT) (s):		
Stride 1	0.39 ± 0.05	0.38 ± 0.04
Stride 2	0.64 ± 0.04 *	0.63 ± 0.06 *
Stride Frequency (SF):		
0–5 yd (Segment 1)	3.75 ± 0.45	3.75 ± 0.45
6–10 yd (Segment 2)	2.92 ± 0.29 ^#^	2.67 ± 0.49 ^#^
Stride Length (SL) (in):		
Stride 1	46.52 ± 3.97	46.19 ± 5.75
Stride 2	52.23 ± 5.36 *	51.54 ± 7.11 *

* Significant difference (*p* < 0.001) between Stride 1 and Stride 2; ^#^ significant difference (*p* < 0.001) between 0 and 5 yd (Segment 1) and 6 and 10 yd (Segment 2); *n* = 12, yd (yard), s (second), in (inch).
